# Ferroptosis Mediated by Lipid Reactive Oxygen Species: A Possible Causal Link of Neuroinflammation to Neurological Disorders

**DOI:** 10.1155/2021/5005136

**Published:** 2021-10-23

**Authors:** Ying Cheng, Yiting Song, Huan Chen, Qianqian Li, Yuan Gao, Guanchao Lu, Chengliang Luo

**Affiliations:** ^1^Department of Forensic Medicine, School of Basic Medicine and Biological Sciences, Soochow University, Suzhou 215123, China; ^2^School of Forensic Medicine, Wannan Medical College, Wuhu 241002, China; ^3^Department of Neurology, Fuping County Hospital, Weinan 711700, China

## Abstract

Increasing evidence indicates a possible causal link between neuroinflammation and neurological disorders, including Alzheimer's disease (AD), Parkinson's disease (PD), Huntington's disease (HD), and stroke. A putative mechanism underlying such a link can be explained by ferroptosis. Current studies have shown that disturbances of iron homeostasis, glutamate excitatory toxicity, lipid reactive oxygen species (ROS), and other manifestations related to ferroptosis can be detected in several neurological disorders caused by neuroinflammation. To date, compelling evidence indicates that damage-associated molecular pattern (DAMP) molecules (e.g., ROS) produced in the process of ferroptosis activate glial cells by activating neuroimmune pathways and then produce a series of inflammatory factors which contribute to neurological disorders. Our review article provides a current view of the involvement of ferroptosis or ROS in the pathological process of neuroinflammation, the effects of neuroinflammation mediated by ferroptosis in neurological disorders, a better understanding of the mechanisms underlying ferroptosis participates in neuroinflammation, and the potential treatments for neurological disorders. In addition, further research on the mechanisms of ferroptosis as well as the link between ferroptosis and neuroinflammation will help provide new targets for treatment.

## 1. Introduction

In 2003, Dolma et al. discovered that camptothecin (CPT) and a new compound erastin could selectively kill tumor cells that expressed RasV12 protein, but their lethal mechanisms are different. Cell death induced by CPT showed changes in nuclear morphology, deoxyribonucleic acid (DNA) fragmentation, and caspase activation, which could be inhibited by caspase inhibitors. However, cell death induced by erastin was different from what had been seen before [1]. Therefore, erastin may have induced a new type of cell death. Later, Yang and Stockwell and Yagoda et al. showed that this mode of cell death could be inhibited by iron-chelating agents, and increased ROS appeared in cells [2, 3]. Dixon et al. officially named this mode of cell death ferroptosis, an iron-dependent and nonapoptotic form of cell death characterized by intracellular accumulation of ROS. Through electron microscopy, it can be observed that the mitochondria of cells with ferroptosis are significantly shrunken with an increased membrane density [4].

Neuroinflammation is a complex process attributing to the interactions of glial cells and peripheral immune cells in the central nervous system (CNS). The activation of glial cells and peripheral lymphocytes releases inflammatory factors that cause damage to the nervous system. Current studies have shown that disturbances of iron homeostasis, lipid ROS accumulation, and other manifestations related to ferroptosis can be detected in some neurological disorders caused by neuroinflammation [5–10], indicating that ferroptosis may play a tremendous role in the occurrence and development of neurological disorders, including Alzheimer's disease (AD), Parkinson's disease (PD), Huntington's disease (HD), and stroke. Therefore, we give a current view on the relationship between ferroptosis and neuroinflammation in neurological disorders, including the putative pathway for ferroptosis that participates in neuroinflammation and the potential treatments for neurological disorders.

## 2. The Pathway of Ferroptosis

**Figure 1:**
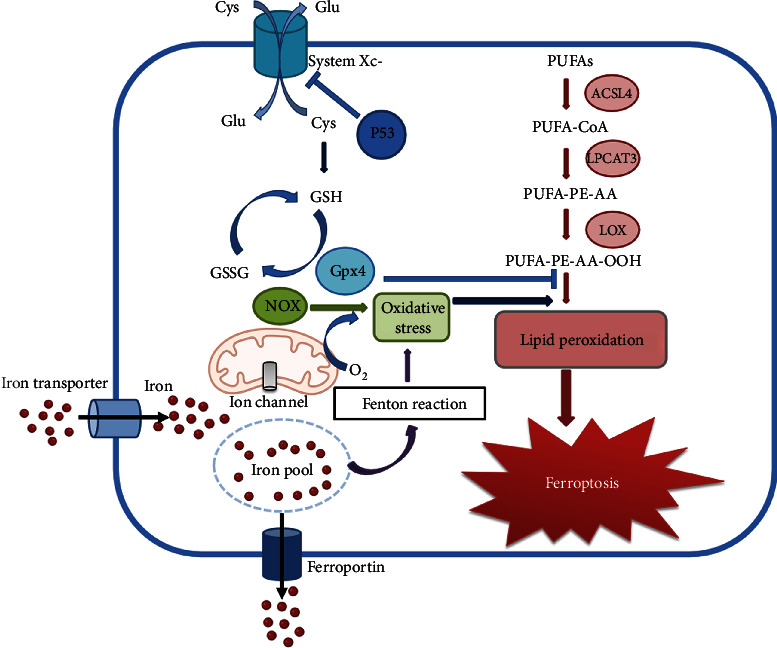
Mechanism pathways of ferroptosis.

As a new type of cell death, ferroptosis is iron-dependent and induced by intracellular lipid peroxidation accumulation. The occurrence of ferroptosis is caused by the imbalance between the production and degradation of lipid ROS in cells. Here, we summarize the main mechanisms involved in the process of ferroptosis such as accumulation of lipid ROS, inhibition of system Xc^−^, glutathione peroxidase 4 (GPX4) enzyme activity, and iron metabolism disorder (Figure 1).

### 2.1. Ferroptosis Mediated by Lipid Reactive Oxygen Species

Apart from the iron-mediated ROS produced by the Fenton reaction, there are plentiful potential sources of ROS in the cell. Among them, one pivotal generator of intracellular ROS is NADPH oxidases (NOX) which is a family of seven transmembrane proteins which transfer electrons across biological membranes, including five monooxidases (NOX1-5) and two dual oxidases (Duox1-2) [11, 12]. When oxygen accepts an electron, the product of the electron transfer reaction is superoxide. The biological function of NOX enzymes is therefore the generation of ROS, including hydroperoxides (H_2_O_2_) [13], which contributes to Fenton reaction. Members of the NOX family have been shown to promote ferroptotic cancer cell death [14–16]. The NOX inhibitor could prevent erastin-induced ferroptotic death [4], whereas NOX activation sensitizes ferroptosis [15]. Mitochondria are another main source of intracellular oxidant production. Electrons are often lost during the transfer of electron transport chain complexes in oxidative phosphorylation of mitochondria. ROS are generated after electrons binding with molecular oxygen, eventually leading to oxidative stress.

As transmembrane channels, voltage-dependent anion channels (VDACs) transport ions and metabolites [17]. Yagoda et al. found that erastin could act on VDACs. With the help of RNA interference (RNAi) technology, it was found that cells with lower expression of VDAC2 or VDAC3 were tolerant to ferroptosis induced by erastin. Furthermore, erastin can also lead to changes in mitochondrial outer membrane permeability. Consequently, Yagoda et al. believed that the interference of VDACs may conduce to mitochondrial dysfunction and ROS release [3].

In addition to NADPH oxidases and mitochondria, extra sources of ROS include plentiful enzymes such as cyclooxygenases (COX), lipoxygenases (LOX), xanthine oxidase (XO), and monoamine oxidase (MAO) which produce ROS because of their enzyme function [18]. Studies have expounded that the polyunsaturated fatty acid (PUFA) chain of membrane lipids can form lipid ROS by reacting with ROS [4]. PUFAs can undergo enzymatic or nonenzymatic oxidation reactions to form lipid H_2_O_2_, which can combine with iron to produce toxic lipid free radicals, causing cell damage. These free radicals can transfer protons adjacent to PUFAs, initiating a new round of lipid oxidation reactions and further transmitting oxidative damage, which directly triggers ferroptosis. Lysophosphatidylcholine acyltransferase 3 (LPCAT3) and acyl-CoA synthetase long-chain family member 4 (ACSL4), two genes related to lipid metabolism, can induce lipid peroxidation. Hence, the suppression of ACSL4 and LPCAT3 may play a fundamental role in inhibiting ferroptosis [19]. Interestingly, Gao et al. found that autophagy may engender ferroptosis by regulating intracellular iron homeostasis and the generation of ROS [20]. Park and Chung also observed that ferroptosis inducer (erastin ) enhanced intracellular lipid peroxidation and ROS in wild-type cells, but not in autophagy defective cells, which indicated that autophagy may induce ferroptosis by increasing lipid peroxidation [21]. What is more, Ma's group demonstrated that iron-mediated ROS generation can induce autophagy [22]. This complex cycle continuously induces ferroptosis and the production of lipid ROS, causing cell damage.

### 2.2. Inducing Ferroptosis by Suppressing System Xc^−^

System Xc^−^, a heterodimer composed of SLC7A11 and SLC3A2 subunits, is an amino acid antiporter widely distributed in the phospholipid bilayer and also well known as an important antioxidant component. System Xc^−^ balances excess hydrogen peroxide and peroxides by regulating the synthesis of glutathione (GSH) through the import and export of cysteine and glutamate [23]. Yang and Stockwell found that the decrease of GSH leads to the decrease in the activity of GPXs, which plays a crucial role in inhibiting the generation of lipid ROS [2], and glutathione is also an essential cofactor that contributes as the main substrate of GPX4 [24]. Pharmacologically, several system Xc^−^ inhibitors have been reported such as erastin, blocking the absorption of cystine, which induces ROS accumulation and ferroptosis [25]. The mRNA and protein expression levels of SLC7A11 were significantly decreased after the upregulation of P53 gene expression, thus confirming that SLC7A11 is a new target of the P53 gene [26].

### 2.3. Inducing Ferroptosis by Suppressing GPX4

There are many members of the GPXs family, including GPX1~GPX8. GPX4 converts reduced glutathione (GSH) to oxidized glutathione (GSSH) and reduces cellular lipid hydroperoxides (L-OOH) to corresponding alcohols [27, 28]. Research has already reported that cells with downregulated GPX4 expression were more sensitive to malnutrition, whereas upregulated GPX4 expression inhibited ferroptosis [2], indicating that GPX4 plays an important role in ferroptosis. In addition, current studies founded that an official ferroptosis inducer (RSL3) and some other compounds, such as DPI7 and DPI10, can directly inhibit GPX4 and reduce the antioxidant capacity of cells. Moreover, the mevalonate pathway which targets GPX4 by regulating the maturation of selenocysteine tRNA gives rise to ferroptosis [29]. Selenocysteine is one of the amino acids in the active center of GPX4, and the insertion of selenocysteine into GPX4 requires a special transporter—selenocysteine tRNA [30]. Maturation of selenocysteine tRNA depends on the transfer of isopentenyl groups from isopentenyl pyrophosphate (IPP) to the precursor of selenocysteine tRNA by isopentenyl transferase [31]. In consequence, the mevalonate pathway can affect the synthesis of selenocysteine tRNA by downregulating IPP and further interfere with the activity of GPX4, resulting in ferroptosis.

### 2.4. Ferroptosis Mediated by Iron Metabolism Disorder

Free iron ions in serum are always transported together with transferrin. The Tf-Fe^3+^ complex binds to the transferrin receptor 1 (TfR1) on the cell membrane and enters the cell through endocytosis, forming a stable endosome. Fe^3+^ in the cells was reduced to Fe^2+^ by the six-transmembrane epithelial antigen of the prostate 3 (STEAP3). Fe^2+^ was transported from the nucleus to the unstable cytosol mediated by divalent metal transporter 1 (DMT1). The excessive free iron ions are stored in ferritin or transferred to the outside of the cell by ferroportin (Fpn) to maintain intracellular iron homeostasis [32]. Our recent study expounded that ferritin-knockout mice are less resistant to ferroptosis [33]. Excess iron can directly catalyze the production of lipid ROS through the Fenton reaction that brings about the continuous accumulation of intracellular lipid ROS and ferroptosis [34]. In addition, non-transferrin-bound iron (NTBI) can be transferred into the cell by ZIP14 and ZIP8 (SLC39A14 and SLC39A8) [35, 36]. Cascades underpinning imbalance of iron metabolism could be active drivers of neuropathology in major neurological disorders.

## 3. The Involvement of Ferroptosis in the Pathological Process of Neuroinflammation

**Figure 2:**
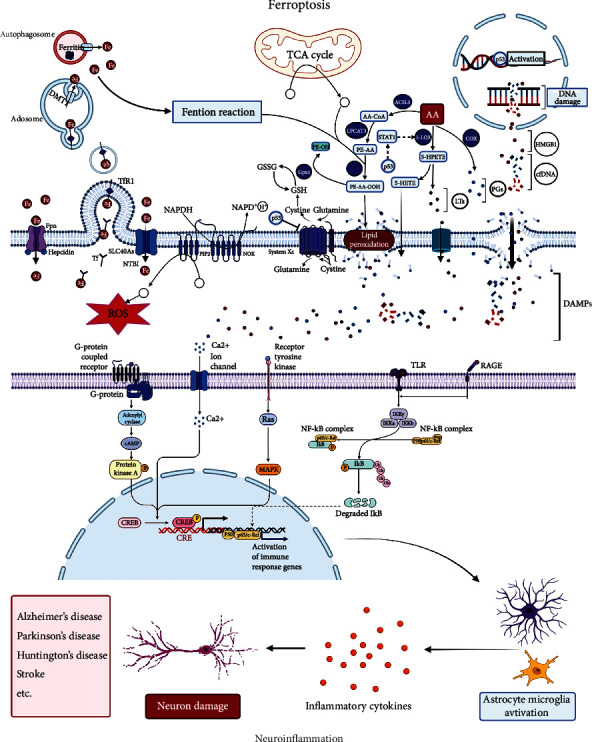
Putative pathway for ferroptosis participates in neuroinflammation to neurological disorders. Damage-associated molecular pattern (DAMP) molecules (e.g., ROS, cfDNA, HMGB1, ITs, and PGs) produced in the process of ferroptosis activate glial cells by activating neuroimmune pathways. Activated glial cells produce a series of inflammatory factors which contribute to neuronal damage and a series of neurological disorders, such as Alzheimer's disease, Parkinson's disease, Huntington's disease, and stroke.

Given that ferroptosis has been discovered to promote pathological processes in neurological disorders, many researchers sought to gain insight into mechanisms of ferroptosis that participates in neuroinflammation to neurological disorders. Summarily, DAMP molecules, produced in the process of ferroptosis, activate glial cells by activating neuroimmune pathways. Activated glial cells produce a series of inflammatory factors which contribute to neuronal damage and a series of neurological disorders (Figure 2).

### 3.1. Activation of NF-*κ*B Pathway by ROS

In the pathogenesis of ferroptosis, a key point is the deficiency of GPX4, which leads to the accumulation of lipid ROS and further bringing about ferroptosis [29, 37]. GPX4 utilizes GSH as a cofactor to remove lipid peroxidation from the membrane. GPX4 reduces the complex hydroperoxide including phosphatidyl hydroperoxide and cholesterol hydroperoxide to the corresponding alcohol to maintain the redox balance and avoid harmful oxidative stress damage. ROS and oxidative stress are common features associated with inflammation [38]. ROS can activate the nuclear factor kappa B (NF-*κ*B) transcription factor system, which is a key player in inflammation-related cancers. The core components of the NF-*κ*B pathway are mainly the inhibitory I*κ*B protein, the I*κ*B kinase (IKK) complex, and the transcription factor NF-*κ*B itself. External stimuli trigger IKK, initiating the phosphorylation, ubiquitination, and degradation of I*κ*B protein. The released dimer of NF-*κ*B is further activated by various posttranslational modifications and is transferred to the nucleus to bind to specific DNA sequences, promoting the transcription of target genes. Studies have shown that ROS, especially H_2_O_2_, can activate IKK in certain cell types [39]. ROS promotes IKK*γ*/NEMO dimerization which is the subunit of IKK by promoting the formation of disulfide bonds between Cys54 and Cys347, resulting in the activation of IKK and the initiation of the NF-*κ*B pathway. Additionally, ROS regulation of the upstream NF-*κ*B activation pathway is also reflected in selective I*κ*B*α* phosphorylation [40]. I*κ*B*α* is usually phosphorylated at Ser32 and Ser36, but H_2_O_2_ causes ubiquitination and degradation of I*κ*B*α* by influencing the phosphorylation of I*κ*B*α* at Tyr42 or other tyrosine residues. The released dimer of NF-*κ*B migrates to the nucleus, where it binds to the *κ*B site on the DNA sequence and promotes the transcription of target genes. Therefore, GPX4 plays a significant role in the elimination of peroxides, reducing the accumulation of ROS and inhibiting the activation of NF-*κ*B. NF-*κ*B is a nuclear transcription factor closely related to inflammation [41], which is expressed in almost all cell tissues, such as microglia, neurons, and astrocytes. It regulates multiple functional target genes and participates in a variety of biological processes such as immune response, inflammatory response, apoptosis, and tumorigenesis. Microglia are one of the most important immune cells in the CNS and play a vital role in both normal brain development and neuroinflammatory diseases. The NF-*κ*B signaling pathway of the glial also plays a critical role in inflammatory responses in the nervous system. Chronic activation of NF-*κ*B can induce transcription of various neuroinflammatory genes [42] and promote the secretion of inflammatory cytokines, such as TNF-*α*, IL-6, IL-1*β*, nitric oxide, adhesion molecules, and neurotoxins, inducing neuronal apoptosis. Accumulated ROS during the ferroptosis process in the brain leads to the activation of the NF-*κ*B pathway in glial cells and the occurrence of neuroinflammation.

### 3.2. Neuroinflammatory Damage Caused by Lipid Peroxidation

In the complex mechanism of ferroptosis, another hallmark lipid peroxidation must be mentioned. Researches have shown that lipids not only form lipid peroxides to interfere with the structures of membrane and functions of membrane proteins but also induce intracellular signal transduction and stimulate other pathways leading to cell death [4, 43]. Many studies have illustrated that lipid may play a vital role in inflammation, immunity, metabolism, and various biofilms. Excessive ROS such as H_2_O_2_ and hydroxyl radical (·OH), as well as reactive nitrogen species (RNS) such as NO· and ONOO-, may give rise to oxidative stress [44], oxidizing biological macromolecules such as lipids, DNA, or proteins. The ·OH is the most dynamic and dangerous reactive oxygen radical, which is formed by H_2_O_2_ in the presence of metal ions. Hydroxyl radicals play a key role in lipid peroxidation, which oxidizes lipids containing carbon-carbon double bonds, especially PUFAs. In PUFAs, the C=C units are separated by a single-bonded C atom, and the hydrogen atoms (diallyl hydrogens) attached to these C atoms (bis-allylic hydrogens) are vulnerable to hydroxyl radical. Therefore, PUFA such as arachidonic acid (AA) is highly oxidized fatty acid. The peroxides of PUFAs and their final production—reactive aldehydes like 4-hydroxynonaldehyde (4-HNE)—lead to carbonylation of proteins, which is associated with atherosclerosis, neurodegenerative diseases, and other diseases [45]. Oxidation of PUFAs can be carried out by nonenzymatic or enzymatic means. Lipoxygenases are ferric enzymes that catalyze the oxygenation of PUFAs to produce fatty acid hydroperoxides. According to the research, the cascade reaction of arachidonic acid involves enzymes related to lipid peroxidation metabolism [46]. The oxidation cascade of AA first releases the polyunsaturated fatty acid AA from the N-2 position of the membrane phospholipid by phosphatase A2 (PLA2). AA produces bioactive eicosanoids (prostaglandins, leukotrienes, and epoxy-fatty acids) using enzymes related to lipid peroxidase (COX, LOX, and cytochrome P450 enzymes). Under physiological conditions, COX-2 plays a role in regulating cerebral vascular and synaptic plasticity in the nervous system. Under pathological conditions, the metabolism of AA mediated by COX to produce prostaglandin was also enhanced in a rat model of focal cerebral ischemia [47]. Prostaglandins, a class of carotenoids, are reported to be a key factor in ischemic and excitotoxic nerve injury. Overexpression of COX-2 during lipid peroxidation mediates neurotoxicity through prostaglandin E2 (PGE2). If the toxic stimulation persists, it may also lead to secondary neuroinflammation damage of the CNS. Experimental studies support that the application of COX-2 inhibitors reduces the level of proinflammatory cytokines such as IL-1*β* and TNF-*α* [48, 49]. Drug suppression or gene knockout of 15-LOX also leads to reduced production of IL-1 and TNF-*α* [50, 51].

### 3.3. DAMPs Inducing Neuroinflammation

Iron disturbance is a basic element of ferroptosis. Excess iron from hemoglobin and the periphery can be transported through a series of ion channels and transporters, including divalent metal transporter 1 (DMT1), transferrin receptor 1 (TfR1), and ferroportin (Fpn). Fe^2+^ directly catalyzes the generation of lipid ROS through the Fenton reaction, resulting in the continuous accumulation of intracellular lipid ROS and causing ferroptosis. It was founded that excess Fe^2+^ transported from the microglia resulted in iron metabolism disorder [34]. In the CNS, glial cells not only continuously monitor the dynamic balance of the internal environment but also act as immune cells, resisting endogenous and exogenous pathogenic stimuli [52]. Immune cells recognize pathogens primarily by pattern recognition receptors (PRRs), which include pathogen-associated molecular patterns (PAMPs) and damage-associated molecular patterns (DAMPs). The main form of PRRs is the Toll-like receptor (TLR) family, which is not only abundant in immune cells but also expressed in CNS glial cells. The danger signal DAMP is an endogenous molecule encoded directly by endogenous genes of the host. It can be released immediately under certain conditions of tissue damage, without the need for protein synthesis. DAMPs from ferroptosis cells and proinflammatory cytokines provide prefeedback signals that reinforce the programmed death of more cells [53]. Severe cycles of death can lead to barrier dysfunction, with inflammation spreading throughout the body, which may eventually bring about extensive multiorgan failure. DAMPs, hemoglobin, erythrocyte, and iron are produced during ferroptosis [54], which can be recognized by PRRs, namely, TLRs in glial cells. Both microglia and astrocytes are activated and secrete inflammatory cytokines such as IFN-*α*, IFN-*β*, IL-1*β*, IL-6, IL-10, IL-12, IL-18, and TNF-*α*, as well as ROS and chemokines, participating in neuroinflammation caused by an infection in the nervous system [55–57]. In the TLR family, the TLR3 signal induces the strongest proinflammatory polarization response, which is characterized by the secretion of high levels of IL-12, TNF-*α*, IL-6, CXCL10, IL-10, and IFN-*β* [58]. In acute infectious or noninfectious CNS diseases caused by ferroptosis of nerve cells, activated TLR signals in glial cells are involved in neuronal injury under the action of endogenous and exogenous pathogens or the release of products from cell damage. In AD, amyloid, S100 protein, and high mobility group box-1 (HMGB1) are secondary key factors following primary neuritis, further exacerbating the production of proinflammatory cytokines. HMGB1 belongs to the Ia DAMP category, which can be perceived by PRRs on several cells. Class Ia DAMPs signal danger to surrounding PRR-carrying cells, such as phagocytes, triggers sterile inflammation [59].

### 3.4. Ferroptosis Mediated by P53 Gene Inducing Neuroinflammation

P53 gene, as a tumor suppressor gene that mediates the inhibition, senescence, and apoptosis of cell cycle, plays an important role in tumorigenesis and development. Recently, acetylated defective P53 mutants have been found to promote ferroptosis [60]. Jiang et al. discovered that the activity of H1299 cells with P53 gene silenced did not change when they were treated with ROS [61]. However, treated with ROS after the P53 gene was activated, 90% of the H1299 cells died, indicating that the activation of P53 reduced the antioxidant capacity of these cells. Studies have demonstrated that the P53 gene mainly affects the synthesis of GSH by inhibiting system Xc^−^ [32]. In addition, P53 can promote ferroptosis by enhancing the expression of spermine N1-acetyltransferase 1 (SAT1), which acetylates spermine and is an important rate-limiting enzyme in polyamine catabolism. The abnormal metabolism of polyamine and aberrant SAT1 expression is associated with a variety of pathological conditions, including neurological disorders and cancer [62, 63]. The activity of SAT1 will increase in a variety of stress reactions, including oxidative stress and inflammatory stimuli. The activation of SAT1 may lead to lipid peroxidation and ferroptosis induced by ROS, resulting in neuroinflammatory damage. Previous studies have also observed that overexpression of SAT1 leads to rapid depletion of spermine in cells, ultimately giving rise to significant growth inhibition and mitochondrial apoptosis [64]. The induction of SAT1 was related to the expression level of 15-LOX, and pharmacological inhibition of 15-LOX by PD146176 attenuated SAT1-mediated ferroptosis [65]. Moreover, 15-LOX, together with COX-2 which is overexpressed during ferroptosis, is also involved in the metabolic process of arachidonic acid, producing prostaglandins, peroxides, ROS, and other substances to induce inflammatory injury of the nervous system [66].

## 4. The Effects of Neuroinflammation Mediated by Ferroptosis in Neurological Disorders

Neuroinflammation of the CNS is an extremely complex process. In the initial stage, glial cells act as immune cells, performing defensive reactions in response to traumatic stimuli. Mediated by ATP, microglia are activated to produce proinflammatory cytokines [67, 68]. Local damage of the CNS may affect other brain regions far away from the damage site through Ca^2+^ waves [69]. Lipid ROS dysregulation can also destroy physiological calcium signals [70]. During the progressive stage of neuroinflammation, the inflammatory microenvironment may induce glial cells to transform into different phenotypes and regulate inflammation through cell feedback and cell-to-cell communication mechanisms, resulting in the proinflammatory and anti-inflammatory reactions of neuroinflammation. Besides, ferroptotic neurons themselves also could release proinflammatory DAMPs, which then triggered the innate immune system in brain tissues [54]. In the prognostic stage of neuroinflammation, glial cells are subjected to endogenous changes such as persistent stimulation, aging, or gene defects under severe pathological conditions, which will lead to chronic neuroinflammation of the CNS and degeneration of neurons. Chronic neuroinflammation is one of the most important mechanisms in the pathogenesis of neurodegenerative diseases. Physiologically, iron, as an important redox metal, produces ATP in the cellular metabolism of the CNS, which supplies energy for cell metabolism in physiological processes. However, when iron metabolism is disrupted, the nerve system is vulnerable to damage caused by excess iron and oxidative stress. Recently, studies supported that iron metabolism disorders, glutamate excitatory toxicity, lipid ROS accumulation, and other characteristics related to ferroptosis can be detected in some neurological disorders. Accumulated evidence demonstrates that ferroptosis participates in the neuroinflammatory pathological process. Ferroptosis inhibitors have also been found to improve the prognosis of neurological disorders. Therefore, further investigation into the mechanism of ferroptosis involved in the neuroinflammatory process will be valuable to the treatment and improvement of neurological disorders. The effects of neuroinflammation mediated by ferroptosis in several characteristic neurological disorders are described below.

### 4.1. Alzheimer's Disease (AD)

Among the classical properties of AD, the amyloid cascade theory is an important study to explain the pathogenesis of AD. Amyloid *β*-protein (A*β*) accumulation is also considered to be an initial event in the pathogenesis of AD. Another feature of AD is intracellular neurofibrillary tangles (NFT) due to hyperphosphorylation of Tau protein. Under physiological conditions, tau protein stabilizes microtubules and regulates axon transport. Pathological changes will make tau separate from microtubules, resulting in synaptic loss and neuronal dysfunction. What is more, the neurocognitive decline is associated with synaptic reduction and neurotransmitter oxidation, which could be attributed to oxidative stress, mainly the increase of ROS and intracellular and extracellular hydrogen peroxide [71, 72]. There has been early evidence of an association between iron accumulation in the cerebral cortex and the development of AD [73, 74]. Svobodová et al. proved that free iron and ferritin accumulated after amyloid plaque formation in the cerebral cortex of *β*-amyloid precursor protein (APP)/presenilin 1 (PS1) transgenic mouse model [75]; iron deposition is involved in the formation of A*β* plaque and the misfolding process of NFT. Inhibition of lipid peroxidation and ferroptosis mediated by ACSL4 and ACSL4 special protein 1 (SP1) mimicked the beneficial effects of aldehyde dehydrogenase (ALDH2) overexpression in APP/PS1-mutant mice [76]. APP is a type 1 transmembrane protein; the proteolytic cleavage of which forms the A*β* is associated with the pathogenesis of AD. In the presence of free iron, A*β* plaques are effectively involved in ROS production, leading to increased lipid peroxidation, protein oxidation, and DNA damage [77]. Proteolysis cleavage in APP occurs through enzyme complexes including *α*-secretase or *β*-secretase and *γ*-secretase. Tsatsanis et al. showed that APP promotes the iron efflux of neurons by stabilizing the expression of ferroportin on the cell surface [78]. The proteolytic cleavage of APP under the action of *β*-secretase leads to the accumulation of iron in the neuron cells, resulting in the generation of A*β*. Hambright et al. found in a mouse model with a condition deletion in neurons of the forebrain of GPX4 that GPX4-deficient mice have significant learning and memory dysfunction, which is related to markers of ferroptosis, such as increased lipid peroxidation and neuroinflammation [79]. Furthermore, treatment with liproxstatin-1, a ferroptosis inhibitor, ameliorated neuronal death and memory impairment induced by A*β* [80]. In addition, enzymes involved in lipid peroxidation, such as COX-2, LOX, and cytochrome P450 enzymes, metabolize AA into substances such as prostaglandins, leukotrienes, and epoxide fatty acids. COX-2 mediates neurotoxicity through PGE2 produced from AA, which is an important member of DAMPs. If the toxic stimulation persists, it may also conduce secondary neuroinflammation damage in the CNS. More and more evidence indicates that the amyloid cascade theory alone may not be convinced to explain the pathogenesis of AD. Therefore, researchers attempt to explore the relationship with other pathological mechanisms. The increased levels of inflammatory markers in the brain of AD patients and the discovery of AD risk genes related to innate immune function suggest that neuroinflammation may play a significant role in the pathogenesis of AD [81–85]. A hallmark of neuroinflammation is the production of proinflammatory cytokines, and the innate immune cells involved in the process of neuroinflammation are mainly microglia and astrocytes. Capillary endothelial cells and permeable blood cells also participate in neuroinflammation, especially when the blood-brain barrier (BBB) is subjected to biochemical or mechanical damage [86, 87]. Astrocytes respond to pathological injury through reactive gliosis, and inflammatory injury induces A1 astrocyte phenotype through the NF-*κ*B pathway, expressing inflammatory mediators. Microglia recognize pathogens, cell debris, or abnormal proteins through TLR and induce microglia cell response [82], internalizing and degrading pathogenic substances through pinocytosis. The process usually disappears once the pathogen or irritant is eliminated. Nonetheless, the function of microglia in the brain of the elderly is impaired. In the case of severe pathological conditions or continuous stimulation by pathogens, the glia is always in the activated state and secreting inflammatory factors, leading to neuroinflammatory damage [88]. Furthermore, crosstalk between glial cells and neurons can also be observed in AD. A*β* activates NF-*κ*B in astrocytes, resulting in an increased release of complement C3, which acts on the C3a receptors of microglia and neurons, and the secretion of IL-1*α* in activated microglia induces the production of A1 astrocytes. In the inflammatory environment of AD, glial crosstalk may form positive feedback, leading to nervous system disorders and self-amplifying inflammatory responses. Evidence from genome-wide association studies also supports the initiating role of neuroinflammation in AD, and mutations in microglia or innate immune genes are associated with an increased incidence of AD in the population [84]. The influence of neuroinflammation on AD has also been confirmed in clinical imaging studies, and the activation of microglia in AD patients is negatively correlated with the structural integrity or functional activity of the brain [89]. Preclinical studies correspondingly support clinical imaging observations that microglia activated by A*β* produce neurotoxic cytokines that lead to neuronal dysfunction [90]. Several current studies have proved that ferroptosis and neuroinflammation play a fundamental role in the pathogenesis and progression of AD, and glial cells are the core of neuroinflammation. Regulating the occurrence of lipid peroxidation and ferroptosis in neurons has important therapeutic significance for neurodegenerative diseases. Meanwhile, targeting glial cells as a breakthrough for drug treatment of AD also has a certain prospect.

### 4.2. Parkinson's Disease (PD)

The typical pathological feature of Parkinson's disease is the degeneration of dopaminergic neurons in the substantia nigra, which is linked with the systematic and progressive accumulation of iron in the substantia nigra, leading to the consumption of dopamine in the striatum. Meanwhile, Lewy bodies also appear in the cells, with *α*-synuclein aggregation as the main component. Studies have shown that the accumulated *α*-synuclein is accountable for the generation of ROS followed by lipid peroxidation in an iron-dependent manner, leading to cell death [70]. The increase of iron in the substantia nigra and globus pallidus in PD patients has also been observed by magnetic resonance imaging (MRI), which is closely related to the duration of disease, neurodegeneration, and the severity of sports injuries [6, 91]. The study indicated that treatment with deferiprone, an iron chelator, substantially reduced unstable iron and biological damage in oxidatively stressed cells, which improve motor function. In addition, studies have shown that the related mechanisms of neuroinflammation, such as activation of microglia, the proliferation of astrocytes, and other peripheral immune cells, may contribute to the impairment of dopaminergic neurons in the substantia nigra. Some cellular and molecular responses related to neuroinflammation may induce harmful events such as oxidative stress and apoptosis mediated by cytokine receptors, eventually leading to dopamine cell death [92]. In 1988, McGeer et al. found activated microglia in the substantia nigra of the nervous system in an autopsy study of patients with Parkinson's disease [93]. Bertrand and colleagues reported that there is mild microglial activation in the locus blue in patients with Parkinson's disease [94]. These are important neuropathological features of Parkinson's disease. Activated microglia produce voluminous oxygen-derived and nitrogen-derived products, among which NO and O^2-^ can combine to form a highly reactive nitrogen species peroxynitrite (ONOO-), which can cause oxidative damage to various proteins. Another important neuropathological feature of the substantia nigra in Parkinson's disease is astrocyte response. Astrocytes may protect the nervous system by eliminating reactive oxygen species in the nervous system through GPX4 or secreting glial cell-derived neurotrophic factor (GDNF). The deficiency or decreased activity of GPX4 may cause ROS accumulation, lipid peroxidation, or activation of the NF-*κ*B pathway to produce inflammatory mediators, resulting in inflammatory damage to the nervous system. There may be few astrocytes around vulnerable neurons in patients with PD, and a limited astrocyte environment may be a susceptibility factor for the disease [95]. Mogi et al. showed increased concentrations of TNF-*α*, TGF-*α*, TGF-*β*1, and IL-1*β* in the striatum of patients with Parkinson's disease. TNF-*α*, IL-1*β*, and IFN-*γ* were also detected in the patient's substantia nigra [96]. In addition, dopaminergic neurons express receptors for these cytokines, which combine with the cytokines to cause neurotoxicity. These inflammatory cytokines, such as TNF-*α*, IL-1*β*, and IFN-*γ*, can induce the expression of nitric oxide synthase (iNOS) or COX-2. LOX and COX are important metabolic enzymes in lipid peroxidation, resulting in the metabolism of arachidonic acid into prostaglandin, leukotriene, and other substances. COX-2 mediates neurotoxicity through PGE2, resulting in inflammatory damage to the nervous system. Concentrations of other enzymes involved in neuroinflammation mediated by oxidative stress are also increased in PD. Biofluid studies also support the role of neuroinflammatory processes in Parkinson's disease, with elevated levels of IL-2, IL-6, and TNF-*α* in the serum of patients with PD. Regardless of the original nerve conduction in PD, prevention or inhibition of ferroptosis in the nervous system, as well as the inflammatory immune response of glial cells, is important to prevent the progression of the disease.

### 4.3. Huntington's Disease (HD)

A study demonstrated that a larger concentration of iron in neurons was observed in a mouse HD model compared to the wild-type model, suggesting that iron accumulation may be involved in neurodegeneration in HD patients. When excessive Fe^2+^ is generated intracellularly, the production of lipid ROS can be directly catalyzed by the Fenton reaction, resulting in the continuous accumulation of lipid ROS and eventually causing cell ferroptosis [34]. Hence, the overexpression of antioxidant genes in HD can be explained as an adaptive response to ROS imbalance. Ferritin is identified as an iron storage protein with antioxidant activity. Previous research reported the accumulation of ferritin in microglia of R6/2 mice appeared to be a protective mechanism to counteract iron accumulation [9]. Mitochondria are the main source of ROS in cells. During the transfer of electron transport chain complexes in oxidative phosphorylation, electrons are often lost, and ROS are generated after electron binding with molecular oxygen, ultimately, leading to oxidative stress. Furthermore, mutated Huntington's protein (mHTT) in HD is expressed in both neurons and glial cells, which can bring about the production of free radicals in the CNS, eventually triggering oxidative stress [97]. The accumulation of reactive microglia and astrocytes can be observed in the brain of HD patients. Upregulated expression of IL-6, IL-8, and tumor necrosis factor (TNF) in cerebrospinal fluid indicates immune activation. Under the condition of the injury, astrocytes magnify the inflammatory process, remove cell debris, and repair the damaged tissue. Astrocytes that express mHTT were found to be functionally deficient, with low expression of glutamate transporters and inhibited glutamate uptake, resulting in striatal neurons being affected by extracellular glutamate and exhibiting the neuronal glutamate excitability associated with ferroptosis observed in HD. In addition, astrocytes expressing mHTT have been shown to reduce the availability of the inflammatory C-C chemokine ligand 5 (CCL5) to neurons [98]. On the one hand, the mHTT reduced the transcription of CCL5 in astrocytes in an NF-*κ*B-dependent manner. On the other hand, it prevents astrocytes from secreting the CCL5 protein; the loss of which may adversely affect neuronal function [99, 100]. The death of neurons and protein aggregates caused by various oxidative stresses, known as DAMPs, can be recognized by PRRs, namely, Toll-like receptors (TLRs) in glial cells, resulting in activation of both microglia and astrocytes and the secretion of inflammatory cytokines, which may cause aseptic encephalitis in HD. Neuroinflammation is an important component of the pathogenesis of HD, and the expression of mutant Huntington protein alters specific processes of glial response, such as cell signaling, cytokine release, and migration. Changes in microglia and astrocyte function may result in a chronic pathogenic inflammatory response that leads to the death of other neurons, leading to more neuroinflammation.

### 4.4. Stroke

Stroke has an extremely high incidence, mortality, and disability, which can be classified into two major categories: hemorrhagic and ischemic stroke according to the original cause of occurrence [101]. Ischemic stroke is caused by the stenosis or occlusion of carotid and vertebral arteries and insufficient blood supply to the brain, resulting in the depletion of oxygen and nutrients, causing a cascade reaction leading to oxidative stress and mitochondrial damage [102]. Researches have indicated that ferroptosis is strongly associated with ischemic stroke. In 2014, Ahmad et al. found that in a mouse model of ischemic stroke, the content of GSH significantly decreased while the content of lipid ROS increased. The imbalance between ROS and antioxidant defense results in oxidative stress, lipid peroxidation, and eventually neuronal damage [103]. Tuo et al. also reported in their research that ferroptosis caused neuronal degeneration after ischemic stroke, and the use of ferroptosis inhibitors such as ferrostatin-1 or liproxstatin-1 exerted the neuroprotection in a mouse ischemic model [104]. Studies have shown that selenium ions can enhance the expression of GPX4 and inhibit ferroptosis by activating transcription factors TFAP2c and Sp1 [105]. The addition of GPX4 in a mouse model of ischemic stroke exerted an effect on the improvement of behavioral defects to some extent. After ischemic stroke, microglia are activated and change morphologically and phenotypically. As the innate immune cells in the CNS, microglia play a key role in the removal of foreign bodies, cell debris, and the production of cytokines. Studies have revealed that the release of cytokines can destroy the blood-brain barrier [102], leading to the aggregation of various inflammatory cells such as macrophages, natural killer cells, and glial cells to the ischemic site to produce inflammatory responses. Interestingly, activated microglia play a dual role in ischemic stroke. On the one hand, microglia cause inflammatory damage to nerve tissue by secreting proinflammatory cytokines, and on the other hand, they also secrete anti-inflammatory factors to play a neuroprotective role [106, 107]. Pharmacological reduction of 15-LOX expression exerted to neuroprotection and improved behavioral recovery in a stroke model [108]. Jin et al. demonstrated that the consumption of microglia by PLX3397, a dual colony-stimulating factor-1 inhibitor, could aggravate cerebral infarction and neurological defects, and the failure of microglia could also exaggerate the expression of inflammatory mediators in the nervous system [109]. Different subsets of microglia may play diverse roles after ischemic stroke in the brain, striking a balance between proinflammatory and anti-inflammatory. Astrocytes, like microglia, also play essential immune functions in the CNS. Under normal physiological conditions, astrocytes take glutamate from extracellular sources. Nonetheless, in severe pathological conditions, such as ischemic stroke, the expression of glutamate transporter EAAT2 in astrocytes is defective, which prevents astrocytes from absorbing excessive extracellular glutamate, giving rise to impaired glutamate excitability of neurons. After ischemic stroke, astrocytes are stimulated by cytokines from neurons and glial cells to generate IL-1*β*, IL-6, and TNF-*α*, which stimulate and exacerbate the inflammatory response.

## 5. Conclusion and Expectation

There are a series of complex biochemical reactions during ferroptosis pathology including lipid ROS accumulation, glutamate transport inhibition, and iron metabolism disorder, which promote neuroinflammation and oxidative stress response and then aggravate the CNS damage. Activated microglia, reactive astrocytes, invading T cells, and overproduction of inflammatory mediators constitute the neuroinflammatory response. In the initial stage, acute inflammatory responses that happen in CNS trauma may play a protective role, limiting the severity of the injury and enhancing neuronal repair. If the acute inflammation does not subside sufficiently, it will turn into chronic inflammation and is difficult to control. In this case, glial cells tend to aggravate oxidative stress on neurons. Although neuroinflammation is not necessary during the initial stage of neurological disorders, a persistent inflammatory response can cause the exacerbation of diseases. Voluminous studies have confirmed that ferroptosis is closely related to AD, PD, HD, stroke, and other neurological disorders. Although the characteristics and pathogenesis of ferroptosis, as well as the pathological mechanism of ferroptosis's involvement in neuroinflammation, have been initially understood, there are still many problems to be solved in this field. For instance, what are the similarities and differences in the molecular mechanisms of ferroptosis in the context of different neurological disorders? The use of inhibitors of ferroptosis in experimental animal models can improve the severity of the diseases, but whether drugs targeting ferroptosis can play a role in the clinical treatment of chronic neuroinflammatory diseases still needs a lot of clinical verification. Further researches on ferroptosis will help us to deeply comprehend the pathogenesis of neurological disorders and provide a theoretical basis and transformation strategies for the clinical prevention and treatment of neurological disorders.
